# Sympathetic involvement in time-constrained sequential foraging

**DOI:** 10.3758/s13415-020-00799-0

**Published:** 2020-05-27

**Authors:** Neil M. Dundon, Neil Garrett, Viktoriya Babenko, Matt Cieslak, Nathaniel D. Daw, Scott T. Grafton

**Affiliations:** 1 Department of Psychological and Brain Sciences, University of California, Santa Barbara, CA 93106 USA; 2 Department of Child and Adolescent Psychiatry, Psychotherapy and Psychosomatics, University of Freiburg, 79104 Freiburg, Germany; 3 Princeton Neuroscience Institute, Princeton University, Princeton, NJ 08540 USA; 4 Department of Experimental Psychology, University of Oxford, Oxford, OX2 6GG UK; 5 Perelman School of Medicine, University of Pennsylvania, Philadelphia, PA 19104 USA

**Keywords:** Decision-making, Sympathetic stress, Learning, Sequential foraging

## Abstract

Appraising sequential offers relative to an unknown future opportunity and a time cost requires an optimization policy that draws on a learned estimate of an environment’s richness. Converging evidence points to a learning asymmetry, whereby estimates of this richness update with a bias toward integrating positive information. We replicate this bias in a sequential foraging (prey selection) task and probe associated activation within the sympathetic branch of the autonomic system, using trial-by-trial measures of simultaneously recorded cardiac autonomic physiology. We reveal a unique adaptive role for the sympathetic branch in learning. It was specifically associated with adaptation to a deteriorating environment: it correlated with both the rate of negative information integration in belief estimates and downward changes in moment-to-moment environmental richness, and was predictive of optimal performance on the task. The findings are consistent with a framework whereby autonomic function supports the learning demands of prey selection.

**Electronic supplementary material:**

The online version of this article (10.3758/s13415-020-00799-0) contains supplementary material, which is available to authorized users.

## Introduction

A specific but nonetheless ubiquitous value-based decision dilemma requires people to approach or avoid sequential offers that pit a reward against an opportunity cost of time, without full knowledge of what offers, if any, may follow. For example, by committing to a specific project, a contract worker receives payment (reward) while eschewing alternative projects during project completion (opportunity time cost), without knowing what alternative options will later emerge.

The Marginal Value Theorem (MVT; Charnov, 1976) proposes an optimality rule for such sequential decisions, whereby yields that exceed the average reward rate (richness) of an environment should be approached and those falling below it should be avoided. The environment and its consequential opportunity time cost thus prescribe choice selectivity; for example, contract workers should only accept projects with a high hourly rate of return (wiring a new supermarket) during construction booms when time has a high opportunity cost, and accept projects with a low hourly rate of return (fixing a faulty domestic appliance) during construction downturns when opportunity time cost is low. Accordingly, studies across various species (Cowie, 1977; McNamara & Houston, 1985), including humans (Hayden, Pearson, & Platt, 2011; Kolling, Behrens, Mars, & Rushworth, 2012), have predicted foraging behavior using MVT inspired models. Recent work in humans further resolves the computational challenge of learning dynamic environmental richness, by demonstrating that sequential choice behavior is best captured by an MVT-inspired learning model. Specifically, decisions to both leave a patch and explore the environment in patch foraging (Constantino & Daw, 2015; Lenow, Constantino, Daw, & Phelps, 2017) and capture behavior in prey selection (Garrett & Daw, 2019) adhere to the MVT-predicted optimality policy that compares yields against fluctuating environmental richness, which is learned via a standard delta rule (Rescorla & Wagner, 1972). This later work suggested that beliefs about environmental richness update with an asymmetric bias, whereby improvements are learned at a higher rate than deteriorations; the “naïve perseverance of optimism” (Garrett & Daw, 2019).

A separate body of literature has investigated how human performance in decision-making and learning tasks varies as a function of stress. Relevant to foraging studies are findings that acute stress levels promote perseveration with devalued stimuli, suggesting a stress-driven shift from goal-oriented to habitual behavior (Schwabe & Wolf, 2009, 2010). However, Porcelli and Delgado (2017) highlight that many studies in this area estimate stress effects with assays of slow-acting glucocorticoids (e.g., cortisol), creating long stress-to-task latency. In the only extant study exploring the relationship between stress-related endocrine activity and foraging behavior, both acute and chronic stress elevation led to overharvesting tendencies in patch foraging (Lenow et al., 2017). The main assays in that study (cortisol and self-report) probed stress fluctuations operating on longer time horizons than the faster-acting learning needed to update beliefs about environmental richness. This time-constant misalignment similarly affects a commonly used alternative assay of putative stress states – galvanic skin conductance – while further confounds such as arousal and spontaneous fluctuations complicate inferences regarding stress-system contribution to pupillometry data (Bradley, Miccoli, Escrig, & Lang, 2008; Joshi, Li, Kalwani, & Gold, 2016; Krishnamurthy, Nassar, Sarode, & Gold, 2017).

Measures of cardiac autonomic physiology have emerged as an exciting new approach for tracking rapid changes in cortically mediated stress responses fluctuating on a trial-by-trial basis, but these have yet to be employed with sequential decision-making tasks. Such measures have nonetheless charted the effects of experimentally manipulated reward and difficulty on summary states of the sympathetic branch of the autonomic system, indexed with aggregated measures of beta-adrenergic myocardial mobilization (reviewed in Richter, Gendolla, & Wright, 2016). Of relevance to sequential decision-making, increased sympathetic states are associated with the difficulty of cognitive tasks and the relevance of reward – i.e., contractility increases with both increased difficulty and increased importance of reward (Kuipers et al., 2017; Richter, Friedrich, & Gendolla, 2008). Further, where task difficulty is either unknown (Richter & Gendolla, 2009) or user-defined (Wright, Killebrew, & Pimpalapure, 2002) – mirroring the adaptive coping situation in sequential decisions – sympathetic states uniquely track reward relevance, suggesting they may be involved with learning the opportunity cost of an environment. Such a link would be supported by recent perceived duration studies reporting a specific association between sympathetic activation and the overestimation of the duration of a painful stimulus (electro-cutaneous stimulation; Piovesan, Mirams, Poole, Moore, & Ogden, 2018). In addition, the association between sympathetic activation and duration overestimation appears to be specific to adaptive events of negative valence, for example, reporting the duration of the presentation of a high-arousal, negative-valence image (a mutilated body) versus a neutral or positive image (Ogden, Henderson, McGlone, & Richter, 2019; van Hedger, Necka, Barakzai, & Norman, 2017). The role of sympathetic activation in learning may therefore further be adaptive, primarily showing associations with environmental deterioration. However, such a conclusion requires linking sympathetic and environmental fluctuations over shorter time-scales.

Here, we employ state-of-the-art cardiac analyses (Cieslak et al., 2018) on electrocardiogram (ECG) and impedance cardiogram (ICG) data recorded continuously while subjects performed a prey selection task, capturing trial-wise modulation of sympathetic contributions of the autonomic state. Using these trial-wise indices, we address three questions; (1) how drive in the sympathetic stress system aligns with choice policy and responds to changes in environmental richness; (2) how activation in this system correlates with learning parameters; and (3) is sympathetic drive associated with optimal task performance. We first describe the general methods regarding participants, task design, physiological recording, and pre-processing. We will then separately describe methods and results for three data analysis branches addressing the above three research questions.

## General methods

### Participants

We recruited 20 subjects via word of mouth. Nine subjects were male and had a mean (standard deviation) age of 19.11 years (1.37) while the remaining 11 female subjects had a mean (standard deviation) age of 20 years (2.18). The total mean (standard deviation) age of our sample (N=20) was 19.65 years (2.18). Two male subjects and one female subject reported themselves as being left-handed. All subjects provided informed consent to participate and experimental procedures were carried out following IRB approval from the University of California, Santa Barbara.

### Statistical power

Continuous cardiac physiology was recorded while subjects performed a task (see below) specifically designed to measure asymmetric (improvement biased) belief updating. Across three previous behavioral experiments (*n* = 40, *n* = 38, *n* = 38), Garrett and Daw (2019) report this asymmetry with large respective effect sizes of *d* = 1.19, *d* = 0.805 and *d* = 0.914 (estimating *d* from reported dependent t-values and sample sizes (Ray & Shadish, 1996)). Our sample size (*n* = 20) is above the minimum level needed (*n* = 12) to replicate this behavioral finding, assuming the true effect is the average of these three observed effect sizes (0.96) with a one-tailed significance of *α* = 0.05 and statistical power of 0.80 (Dhand & Khatkar, 2014). Note that this assessment does not relate to the power of effects involving cardiac activity.

### Task

**Fig. 1:**
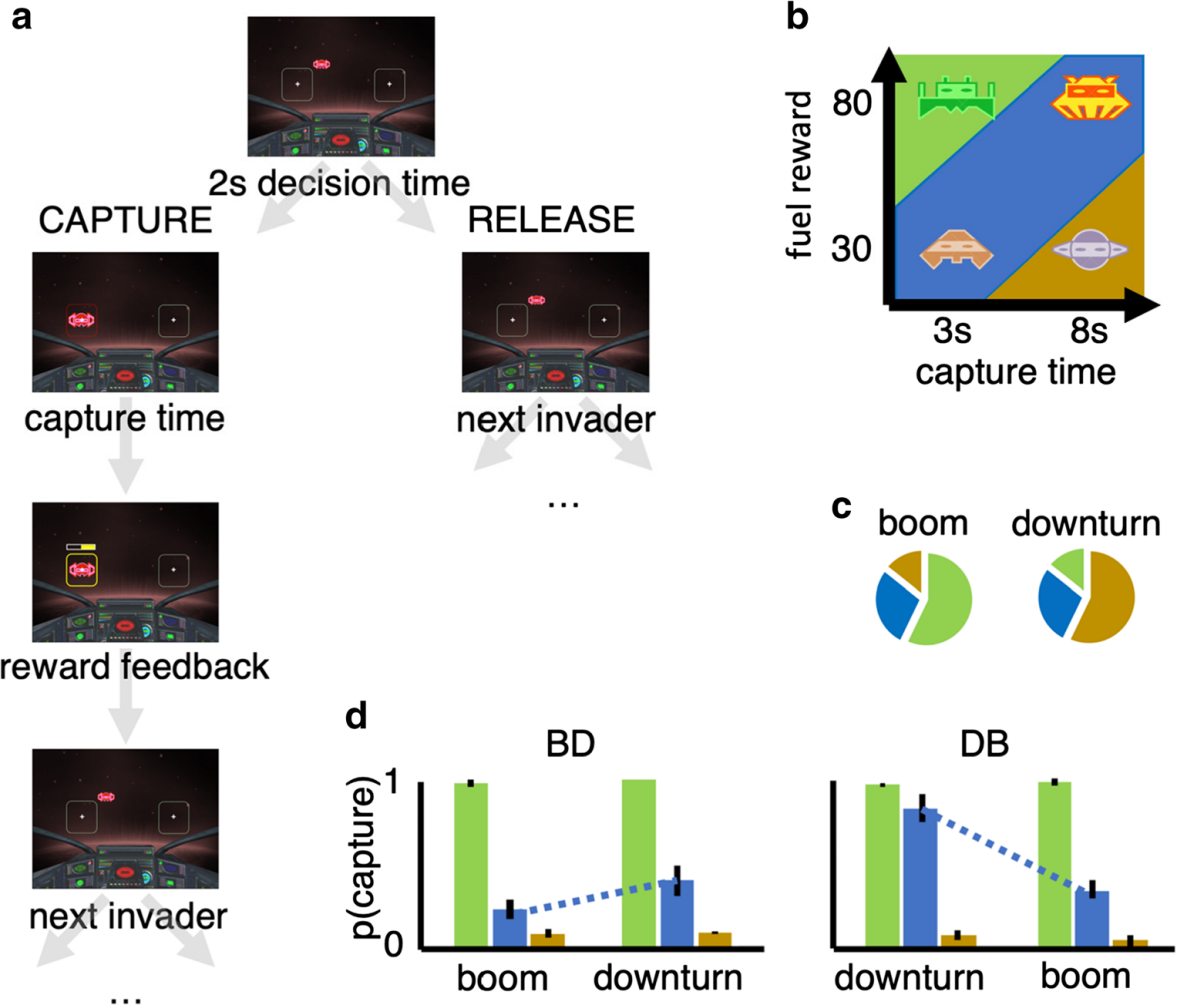
Prey selection paradigm. Subjects decide whether to capture or release serially approaching invaders during their 2-s approach to the cockpit (**panel A**). Releasing an invader progresses immediately to the next invader, while capturing the invader incurs a capture time cost, and fuel reward. Four invader identities (**panel B**) map onto a two-by-two reward-by-cost value space, and can be described categorically as high (green), mid (blue), or low (brown) profitability. Subjects foraged for 12 min in each of two environments (**panel C**) with different proportions of invader profitability. **Panel D:** Replication of Garret and Daw (2019) learning asymmetry. Order of foraging (boom – downturn, BD; downturn – boom, DB) predicts optimal behavior (higher rank 3 captures in downturn relative to boom state). Learning deterioration of an environment takes longer than learning state improvement. Error bars illustrate the standard error of the mean across subjects

Subjects spent 24 min playing the Prey Selection task (Garrett & Daw, 2019), a computerized video game emulating a formal sequential foraging task under a time constraint (see Fig. 1A-C). Subjects were pilots of a space ship and instructed to harvest as much fuel as possible to earn a bonus ($0.01 per point). Fuel was harvested by capturing sequentially approaching space-invaders. Invaders carried either a high (80 points) or a low (30 points) fuel reward, and a high (8 s) or a low (3 s) capture cost. The four identities (see Fig. 1B) mapped onto a three-tier profitability rank: high (high reward/low cost); mid (high reward/high cost, or low reward/low cost); and low (low reward/high cost). Participants spent half of the game-time foraging in an environment with a disproportionately high concentration of high profit invaders (boom; high:mid:low = 4:2:1) and the other half in an environment with a disproportionately high concentration of low profit invaders (downturn; high:mid:low = 1:2:4) (see Fig. 1C). The two environments had different background colors, and subjects were informed that they would differ in terms of invader concentrations, but not explicitly how. Half of the subjects foraged in the order boom to downturn (BD) and the other half in the order downturn to boom (DB), with an opportunity to rest in between the two environments.

Subjects performed the Prey Selection task seated 150 cm from a 68.6-cm (diameter) computer monitor and registered responses on a standard PC keyboard. Experimental stimuli were presented on a Mac mini-computer, using Psychtoolbox extensions (Brainard and Vision, 1997; Kleiner, Brainerd & Pelli, 2007; Pelli, 1997) in MATLAB v9.4 (MATLAB, 2018).

The task paradigm is described in Fig. 1A. At all times subjects saw a cockpit with two target boxes, one on the left and one on the right of the screen. On each trial, subjects had 2s to decide if they wished to capture or release an invader approaching their cockpit. Invaders would pseudo-randomly approach one of the two target boxes. Captures were registered by holding the response button corresponding to the target box in the path of the incoming invader (z with left index finger for the box on the left, m with right index finger for the box on the right). Subjects were required to keep holding the response button as the invader finished its 2s approach to the box, and thereafter for the entirety of the capture time (2s or 7s). Following a successful capture, a feedback screen (1s) described the harvested reward, during which subjects could release the response button. After the feedback screen the next invader immediately began its approach. Releases were registered by holding the response button corresponding to the response box opposite the incoming invader. Subjects were required to hold the response button until the invader reached the capture box. The invader would then disappear and the next invader would begin its approach. Errors carried an 8s time penalty, during which the response boxes disappeared and no invaders approached. Errors were (1) failing to register any response during the 2s invader approach; (2) releasing the response button before the invader reached the response box (captures and avoids); (3) releasing the response button before the end of the capture cost (captures only). On average, participants made an error on 1.82% of trials (standard deviation, 1.00%). Note that requiring the full approach time on both captures and releases, regardless of the latency of response execution, subjects can only use choice policy and not vigor (see Guitar-Masip et al., 2011) to optimize performance. Also, trial-wise pseudorandom mapping of capture and release onto either hand reduced the confounding influence of action hysteresis on decisions (see Valyear et al., 2019). Finally, to encourage full exploration of each environment, 25% of trials were forced-choice. On these trials, a red asterisk would appear above one of the two capture boxes, and participants were instructed to press this response button regardless of whether they wished to perform the corresponding capture or release. Error and forced-choice trials were excluded from later analyses. Participants received a standardized set of instructions, performed a 2-min block of practice trials, and required a perfect score on a questionnaire probing task comprehension before starting the task.

### Physiological recording and preprocessing

Physiological measures of ICG and ECG were collected using non-invasive approaches with a total of ten EL500 electrodes. Prior to each electrode placement, an exfoliation procedure was performed on each electrode location to maximize signal quality. An approximate 1-in. area of skin was cleaned with an abrasive pad, followed by exfoliation with NuPrep gel (ELPREP, BIOPAC, n.d). Once the skin area was fanned dry, a small amount of BIOPAC GEL100 was placed on the electrode and on the skin. In order to assess ICG, a total of eight of the ten electrodes were placed on the neck and torso: two on each side of the neck and two on each side of the torso (Bernstein, 1986). ECG recordings were obtained with a total of two sensors placed beneath the right collarbone and just below the left ribcage. The ICG electrodes provided the necessary ground. Continuous ECG was collected using an ECG100C amplifier and continuous ICG using a NICO100C amplifier (both from BIOPAC). Data were integrated using an MP150 system (BIOPAC) and displayed and stored using AcqKnowledge software version 4.3 (BIOPAC). Both ECG and ICG timeseries were recorded at 1,000 Hz. We recorded raw ECG (ECG) and both the raw (z) and derivative $\left(\frac{dz}{dt}\right)$ of the ICG; the latter facilitates the identification of key impedance inflection points required to estimate the pre-ejection period (PEP). Both z and $\frac{dz}{dt}$ were high-pass filtered to remove respiratory artefact. Below, reference to continuous ICG refers to $\frac{dz}{dt}.$

**Fig. 2:**
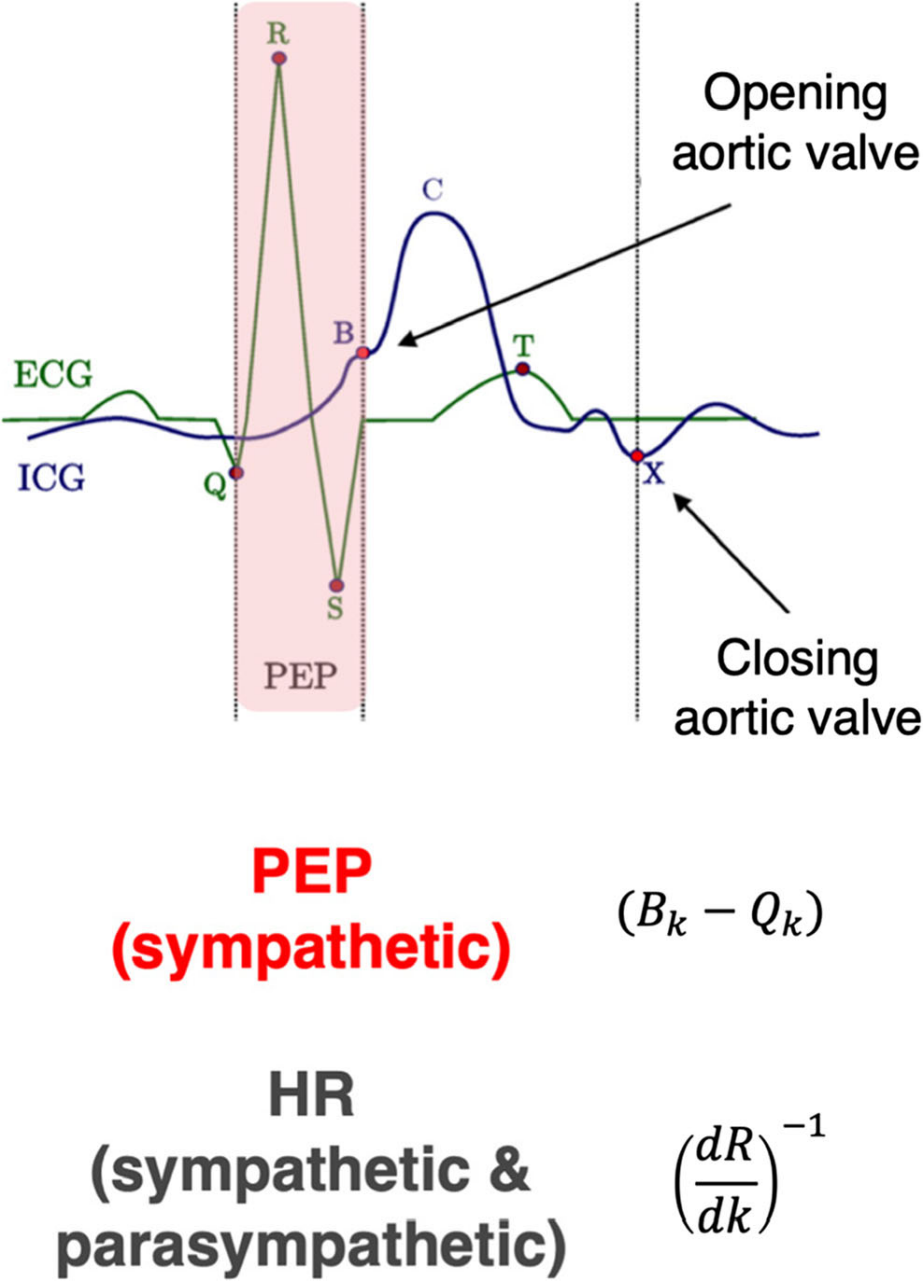
Dynamics of a template heart beat (k), as measured by electrocardiogram (ECG; green) and impedance cardiogram (ICG; blue). Pre-ejection period (PEP) indexes sympathetic-mediated myocardial contractility, computed as the time between early ventricular depolarization (point Q on the ECG) and the opening of the aortic valve (point B on the ICG). Note that in our analyses we used a more easily identified ECG landmark for early ventricular depolarization (point R) and reverse-signed each estimate (see *Methods:**Physiological recording and preprocessing*). Heart rate (influenced by both sympathetic and parasympathetic activity) is computed as the reciprocal of the R-R intervals

We extracted an estimate of the sympathetic state at each heartbeat (see Fig. 2) – pre-ejection period (PEP). Semi-automated software MEAP labeled the continuous ECG and ICG (Cieslak et al., 2018). For each heartbeat, the ECG R point serves as the t=0 landmark for within-heartbeat events. The time interval between the ECG Q point and the ICG B point (see Fig. 2) defines the pre-ejection period (PEP) of a heartbeat, which is related to the contractility of the heart muscle before blood is ejected. However, due to difficulty in reliably capturing the relatively small Q point, PEP is often calculated as the difference between the easily detected ECG R point and the ICG B point (the RBI). This latter interval is comparable to PEP in reliability (Kelsey et al., 1998, Kelsey, Ornduff, & Alpert, 2007) and validity (Kelsey et al., 1998; Mezzacappa, Kelsey, & Katkin, 1999) and sometimes referred to as PEPr (Berntson, Lozano, Chen, & Cacioppo, 2004). We used the RBI definition for our measure of PEP. Reduced values of PEP reflect a shorter pre-ejection interval, indicating increased sympathetic cardiovascular drive. However, to align PEP fluctuations with increases in sympathetic activity, allowing easier apprehension of results of later analyses, we negative signed (i.e., *-1) all extracted values. For all subsequent references to PEP, higher values reflect increased sympathetic cardiovascular drive.

Finally, we used the reciprocal of R-R intervals as a measure of heart rate (HR), such that higher HR values reflect a decrease in the interval between R points, i.e., increased heart rate. Heart rate is influenced by both sympathetic and parasympathetic inputs. We included it in our analyses to control for a known cardiac effect where increased left-ventricular preload time (which occurs with slowing HR) can shorten PEP independent of sympathetic influences (Sherwood et al., 1990).

Trial-wise estimates of PEP and HR were next derived by taking an average from all heartbeats during the 2s time window while invaders approached the spaceship on each trial. This first provided trial-wise estimates of the physiological states during a uniform length time window for all trials, regardless of the executed decision or the identity of the invader, and further allowed us to capture multiple samples, and more resolute evidence, of the physiological state on each trial.

Finally, each trial-wise physiology estimate was corrected again for trial-wise respiratory state. We performed this additional trial-wise respiration correction to account for known influences of respiratory activity on heart rate (Larsen, Tzeng, Sin, & Galletly, 2010), computed in our pipeline from raw ECG R points. Both the magnitude and phase of continuous respiratory activity can be directly estimated from the magnitude of the low-passed (<0.30 Hz) cardiac impedance time series (i.e., low-passed z time series, from above). We then defined trial-wise respiratory state as the average normalized product of the phase and magnitude of respiration activity at each R point during the 2-s time window of invader approaches. Respiration-corrected measures of each trial-wise physiological state were the residuals from a linear model of each raw trial-wise physiology state and the trial-wise respiratory state, performed separately for each subject.

We now present the statistical analysis methods and results separately for three separate branches of analyses. The first branch uses ANOVA and linear mixed-effects models to explore the dynamics between PEP and HR, choices and objectively estimated measures of environmental richness and its moment-to-moment derivatives. The second analysis employs computationally modelled subjective estimates of the environment’s richness, and compares models that allow the learning parameter to vary with PEP and HR. In the third and final analysis we test if blockwise changes in PEP or HR predict optimal task performance.

## Methods - Analysis branch 1: Sympathetic stress, choices, and environment dynamics

All ANOVAs and trial-wise mixed-effects models were fitted using lme4 (Bates, Mächler, Bolker, & Walker, 2014) and lmerTest (Kuznetsova, Brockhoff, & Christensen, 2017) packages in R. Unless otherwise specified, trial-wise logistic models were fitted with logit link functions and Laplacian maximum likelihood approximation, while trial-wise models of continuous measures used restricted maximum likelihood approximation (REML). To ensure model convergence, each trial-wise mixed-effects model used a random intercept model, i.e., fitted a fixed effect for each specified coefficient, and an individual intercept for each subject. When reporting significant model coefficients, we further report the mean and standard deviation of the distribution of the relevant coefficient, re-running the model *n* times using leave-one-out jack-knife resampling of the *n* subjects (respectively: *β*_*μ*_ and *β*_*σ*_). For both ANOVA and trial-wise mixed-effects models, significant lower order marginal effects of significant higher-order interactions are not reported in the main text (however, see Supplementary Tables for a summary of all effects). Post hoc ANOVA contrasts use Tukey correction. All other data pre-processing and analyses were conducted using MATLAB v9.4 (MATLAB, 2018).

## Results - Analysis branch 1: Sympathetic stress, choices, and environment dynamics

Our first behavioral analysis attempted to replicate the Garrett and Daw (2019) finding regarding asymmetric belief updating. To this end, we ran a three-way mixed ANOVA of mean capture-rate as a function of between-group factor *order* (RP, PR) and two repeated-measures factors *env* (boom, downturn) and *rank* (hi, intermediate, low). Summarized in Fig. 1D, the ANOVA reported a significant three-way interaction between *order*, *env* and *rank* (*F* = 3.97, *df* = (2, 90), *p* = 0.022). We accordingly contrasted mean capture rates between BD and DB for the six levels of the env*rank interaction. These contrasts demonstrated significantly higher capture of mid-rank invaders in the downturn environment for order DB, relative to BD (*μ* = 0.828 vs. *μ* = 0.402, both *s*. *e*. = 0.050, *p* < 0.001), with no other contrasts reaching statistical significance. In other words, foraging during the two order conditions differed only in terms of adjustments in the number of mid-rank captures in the downturn environment, in line with Garrett and Daw (2019), and suggestive of slower learning in the face of a contextual deterioration (see supplementary analyses for similar results replacing *rank* (hi, intermediate, low) with a four-level variable corresponding to the four specific reward/cost combinations of the invaders (i.e., hi/low, hi/hi, lo/lo, lo/hi).

A corollary of asymmetric belief updating (prioritizing positive information) is a decision threshold weighted preferentially toward recent reward over recent cost. To formally probe the influence of current and recent offers on choice behavior, we fitted a trial-wise mixed-effects model of *choice*_*t*_ (capture, release) as a function of an intercept term and four parameters: reward and delay on a given trial *t* (respectively: *reward*_*t*_, *delay*_*t*_) and reward and delay on the previous trial, i.e., *t* − 1 (respectively: *reward*_*t* − 1_, *delay*_*t* − 1_). The model yielded a significant positive influence of *reward*_*t*_ (*β* = 2.07, *s*. *e*. = 0.065, *p* < 0.001, *β*_*μ*_ = 2.07, *β*_*σ*_ = 0.071) and significant negative influence of *delay*_*t*_ (*β* =  − 2.05, *s*. *e*. = 0.066, *p* < 0.001, *β*_*μ*_ =  − 2.05, *β*_*σ*_ = 0.068) on capture probability. Choice was also influenced by reward on the previous trial; *reward*_*t* − 1_: (*β* =  − 0.287, *s*. *e*. = 0.064, *p* < 0.001, *β*_*μ*_ =  − 0.288, *β*_*σ*_ = 0.018), but not by the previous delay; *delay*_*t* − 1_: (*β* = 0.071, *s*. *e*. = 0.064, *p* = 0.353). The negative coefficient for *reward*_*t* − 1_ is predicted by MVT; for example, a high reward drives positive belief updating (of the environment’s richness), increasing opportunity cost to future captures, and decreasing future acceptance. The specific influence of *reward*_*t* − 1_ further supports the Garrett and Daw (2019) finding that positive information integrates more readily into state appropriate behavioral policy, relative to negative information.

We next assessed the influence of current physiological state on the relationship between current offer and choice behavior. In two separate models, i.e., one each for PEP and HR, we ran a trial-wise mixed-effects model of *choice*_*t*_ (capture, release) as a function of the two-way interaction effects *phsyiology*_t_*reward_*t*_ and *physiology*_t_*delay_*t*_. We also included, in each model, an intercept term, and nuisance coefficients *order* (BD, DB), *env* (boom, downturn) and *trial index*. In line with the behavior analyses above, each model showed the same influence of *order* and *state* on choice – higher capture rates in the downturn state, and for the DB order (all p-values <0.004). Each model also returned a significant negative coefficient for *trial index* (all p-values <0.001) reflecting higher capture rates early in foraging.

In addition, the PEP model returned a significant positive coefficient for the physiology*delay effect (*β* = 0.461, *s*. *e*. = 0.133, *p* < 0.001, *β*_*μ*_ = 0.460, *β*_*σ*_ = 0.101), suggesting that increased sympathetic cardiovascular drive (indexed by PEP) blunts the negative association between delay and capture. The physiology*reward coefficient did not reach statistical significance (*β* =  − 0.200, *s*. *e*. = 0.132, *p* = 0.128).

The HR model also returned a significant negative coefficient for the physiology*delay effect (*β* =  − 0.703, *s*. *e*. = 0.152, *p* < 0.001, *β*_*μ*_ =  − 0.710, *β*_*σ*_ = 0.151), and a significantly positive coefficient for the physiology*reward effect (*β* = 0.922, *s*. *e*. = 0.152, *p* < 0.001, *β*_*μ*_ = 0.915, *β*_*σ*_ = 0.161), suggesting that decreased heart rate blunts both the aversion of delay and the appeal of reward.

The findings from these preliminary models suggest that increased sympathetic activation is aligned with lower value acceptance, specifically to the cost dimension of value. HR, in contrast, decelerates during moments of low value capture. A shortcoming of these models, however, is that they do not consider value relative to the current rate of reward, i.e., environmental richness. Also, by running two separate models, i.e., one each for the two different physiology variables, we cannot confirm if physiological associations with reward and delay are independent of one another. We accordingly simplified the parameter space such that a single trial-wise parameter ${\ddot{value}}_t$ would account for both dimensions of value, adjusted by an evolving estimation of the opportunity cost at the time of each choice.

$\ddot{value}$for a given trial *t* was defined as:

1 $$ {\ddot{value}}_t={reward}_t-{\theta}_t $$

Where *reward*_*t*_ is the reward of the offer on trial *t* and *θ*_*t*_ is the opportunity cost of capturing offer *t*,computed as:

2 $$ {\theta}_t={delay}_t\ast {\mu}_t $$

Where *μ*_*t*_ is the average rate of reward captured per second through to the beginning of trial *t*, i.e. where *t* > 1, *μ*_*t*_ is updated with:

3 $$ {\mu}_t=\frac{\left({\mu}_{t-1}\ast {s}_{t-1}\right)+\left({r}_{t-1}\ast {c}_{t-1}\right)}{s_t} $$

where *c*_*t* − 1_ is a discrete variable reflecting the selection for trial *t* − 1 (1 = capture, 0 = release), *r*_*t* − 1_ is the reward yielded from capturing the invader for trial *t* − 1 and *s*_*t*_ *and s*_*t* − 1_ are, respectively, the time in seconds of the first frame of trials *t* and *t* − 1 (relative to opening frame of the first trial of the experiment). To ensure early trials did not impart disproportionate influence in the model, *μ*_*t*_ is initialized for all subjects for *t* = 1 in the first block as the average reward rate harvested during the entire experiment across all subjects (7.97 points per second), while *μ*_*t*_ is initialized for all subjects for *t* = 1 in the second block as their individual average value of *μ*_*t*_ in the first block.

Accordingly, $\ddot{value}$ approaching positive ∞ reflect offers that exceed the opportunity cost of the current moment, and should readily be captured, while $\ddot{value}$ approaching negative ∞ describe readily avoidable offers where the reward harvested in exchange for the absorbed time cost is not justified at that moment. In choice models, this relationship is reflected by a positive coefficient, i.e., higher choice probability follows higher $\ddot{value}$.

**Fig. 3:**
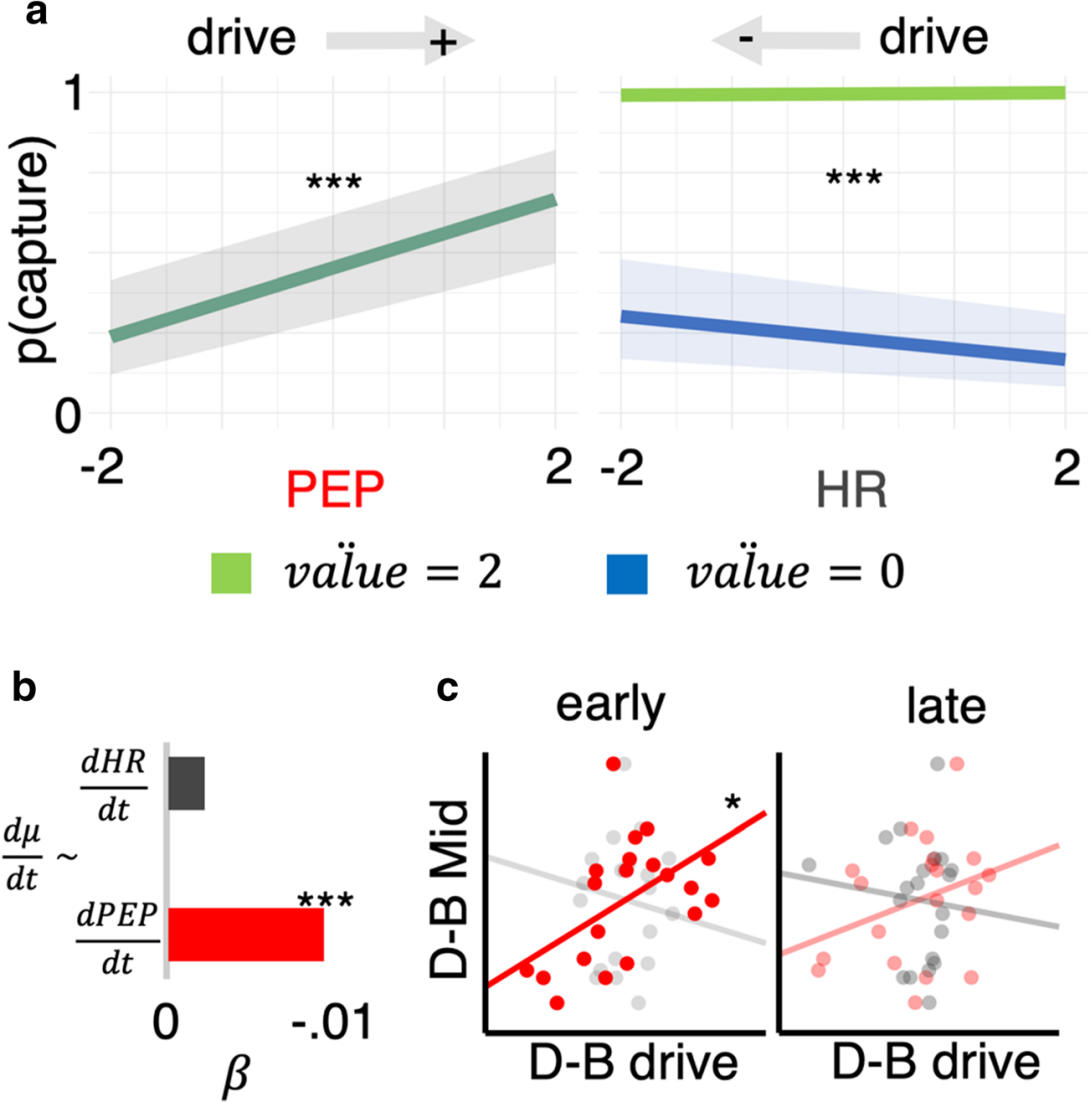
Relationship between autonomic states, value and capture. **Panel A:** A single model of trial-wise choice returns a significant main effect where increased contractility (shorter PEP) increases capture (left panel) regardless of value and a significant interaction where increased low value capture is associated with decreased heart-rate (HR). Here, value is described as in Eq. [1], i.e., reward relative to opportunity cost in current objective rate of reward, with two levels of the continuous value (0 = low; 2 = high) modelled for illustration. As a guide, gray arrows above the plot describe direction of increased (+) or decreased (-) drive within the respective physiological variable. **Panel B:** PEP tracks deterioration in environmental richness. Only trial-wise changes in PEP $\left(\frac{\mathrm{dPEP}}{\mathrm{dt}}\right)$ significantly predicted trial-wise changes in environmental richness ($\frac{\mathrm{d}\upmu}{\mathrm{d}\mathrm{t}}$), negative coefficient indicates that decreases in reward rate, i.e., deterioration, increased contractility (shorter PEP). **Panel C:** PEP predicts optimal learning. Blockwise changes in percentage of rank 3 captures (downturn – boom) modeled as a function of blockwise changes in PEP (red) and HR (gray); separately for mean physiological changes in the early (0–360 s) and later (360–720 s) portion of blocks. Optimal performance (higher D-B rank 3 score) predicted by higher relative drive in early downturn state, relative to early boom, only for PEP. *p<0.05; ***p<0.001

We then ran a single model of *choice*_*t*_ (capture, release) as a function of two two-way interaction effects: *PEP*_t_*${\ddot{value}}_t$ and *HR*_t_*${\ddot{value}}_t$, in addition to an intercept term, and nuisance coefficients *order* (BD, DP), *env* (boom, downturn), and *trial index*. The model returned a negative coefficient for the $PEP\ast \ddot{value}$ interaction, which did not reach statistical significance (*β* =  − 0.188, *s*. *e*. = 0.112, *p* = 0.092). However, a main effect of *PEP* reached significance (*β* = 0.465, *s*. *e*. = 0.128, *p* = 0.000, *β*_*μ*_ = 0.465, *β*_*σ*_ = 0.053). The $HR\ast \ddot{value}$ interaction reached statistical significance (*β* = 0.708, *s*. *e*. = 0.108, *p* < 0.001, *β*_*μ*_ = 0.718, *β*_*σ*_ = 0.151), indicating that a slower heart-rate blunts the positive association between value and capture (see Fig. 3a).

The findings from these initial models suggest sympathetic drive has limited involvement with trial-wise choice in context, with slowed-heart rate (influenced by different branches of the autonomic system) primarily aligned with capturing low value in context. We next tested whether PEP or HR were associated with derivatives (perturbations) of rate of reward, i.e., learning the richness of the environment. We consider *μ*_*t*_ from Eq. [3] a proxy for the evolution of this reward rate. The derivative of *μ*_*t*_ with respect to trial *t*, i.e., ${\frac{d\mu}{d t}}_t$, represents trial-wise perturbations. We modelled ${\frac{d\mu}{d t}}_t$ as a function of ${\frac{dPEP}{dt}}_t$ and ${\frac{dHR}{dt}}_t$. The model also contained an intercept term and nuisance coefficients *env*, *choice*, *order*, and *trial index*.

This model of $\frac{d\mu}{d t}$ (see Fig. 3B) returned a significantly negative coefficient for $\frac{dPEP}{dt}$ (*β* =  − 0.008, *s*. *e*. = 0.002, *p* < 0.001, *β*_*μ*_ =  − 0.008, *β*_*σ*_ = 0.0007), indicating countercyclical perturbations in contractility and reward rate – i.e., if the reward rate decreased, contractility would increase, and vice versa. The coefficient for *HR* failed to reach statistical significance (*β* =  − 0.002, *s*. *e*. = 0.002, *p* = 0.438). Perturbations in the reward rate are therefore exclusively associated with sympathetic drive; contractility increases as reward rate reduces. We next probed whether the $\frac{dPEP}{dt}$ effect was specifically due to sympathetic engagement in environmental deterioration, or disengagement in environmental improvement. We separately ran the model of $\frac{d\mu}{d t}$ on two sets of trials, one where $\frac{d\mu}{d t}$ was negative, i.e., deterioration (*n* = 3,063), and one where $\frac{d\mu}{d t}$ was positive, i.e., improvement (*n* = 3063). Sympathetic engagement only correlated with environmental perturbations when the rate of reward decreased (*β* =  − 0.012, *s*. *e*. = 0.003, *p* < 0.001, *β*_*μ*_ =  − 0.013, *β*_*σ*_ = 0.0009), in the same countercyclical manner as before, while no significant relationship between $\frac{dPEP}{dt}$ and $\frac{d\mu}{d t}$ was observed on the set of trials reflecting environmental improvement (*β* = 0.003, *s*. *e*. = 0.003, *p* = 0.262).

Finally, we reran all models in Analysis branch 1 using the raw physiology measures, i.e., not corrected for respiration state using the residualization procedure outlined in the pre-processing section. The pattern of results was the same across all models.

## Methods - Analysis branch 2: Sympathetic stress and learning parameters

The key finding from our analysis so far centers on perturbations in the sympathetic state tracking perturbations in the reward rate. This may reflect a relationship between the sympathetic state and learning; however, the objective measure of reward rate used above (the rate of reward harvested per second) is not direct evidence that subjects are learning these perturbations in environment quality. In this branch of analysis, we employ computational models fitted to the choice data, and estimate parameters of the subjective estimate of reward rate, in addition to parameters scaling the learning of perturbations in the reward rate. Recall from the previous section, that *μ*_*t*_ represents a moving threshold against which encountered options can be assessed. A simple means by which participants can keep track of *μ*_*t*_ (Constantino & Daw, 2015; Hutchinson, Wilke, & Todd, 2008; McNamara & Houston, 1985) is to implement an incremental error-driven learning rule (Schultz, Dayan, & Montague, 1997; Sutton & Barto, 1998) whereby a subjective estimate of *μ*_*t*_, which we will refer to here as *ρ* for clarity, is incrementally updated according to recent experience. The optimal policy from the MVT remains the same: capture an option *i*, whenever the reward, *r*_*i*_, exceeds the opportunity cost of the time taken to pursue the option. As with Eq. [2], opportunity cost is calculated as the time, *delay*_*i*_, that the option takes to pursue (in seconds) multiplied by the subjective estimated of the reward rate, *ρ*.

4 $$ {c}_i={delay}_i\ast \rho $$

We refer to this measure of opportunity cost, using the subjective estimate *ρ*, as *c*_*i*_. As with the objective measures, participants should capture items that exceed their subjective estimate of the reward rate, i.e., whenever *r*_*i*_≥ *delay*_*i*_ ∗ *ρ***.** Note that we assume quantities *r*_*i*_ and *delay*_*i*_ are known to participants from the outset since they were easily observable and each of the four invader identities (*i* = {1, 2, 3, 4}) always provided the exact same *r*_*i*_ and *delay*_*i*_.

We assumed that subjects learn *ρ* in units of reward, using a Rescorla-Wagner learning rule (Rescorla & Wagner, 1972; Sutton & Barto, 1998), which is applied at every second (*s*). After each second, their estimate of the reward rate updates according to the following rule:

5 $$ \rho \left(s+1\right)=\rho (s)+\alpha \ast \delta (s) $$

Here, *δ*(*s*) is a prediction error, calculated as:

6 $$ \delta (s)=r(s)-\rho (s) $$

where *r*(*s*) is the reward obtained. *r*(*s*) will either be 0 (for every second in which no reward is obtained, i.e. during approach time, capture time, and timeouts from missed responses) or equal to *r*_*i*_ (following receipt of the reward from captured option *i*).

The learning rate *α* acts as a scaling parameter and governs how much participants change their estimate of the reward rate (*ρ*) from one second to the next. Accordingly, *ρ* increases when *r*(*s*) is positive (i.e., when a reward is obtained) and decreases every second that elapses without a reward.

### Symmetric and asymmetric models

We first implemented two versions of this reinforcement learning model, used previously to test for the presence of learning asymmetries in this task (Garrett & Daw, 2019): a *Symmetric Model*, with only a single *α* and a modified version, an *Asymmetric Model*, which had two *α*s: *α*^+^*and α*^−^. In this second model, updates to *ρ* apply as follows:

7 $$ \rho \left(s+1\right)=\rho (s)+\left\{\begin{array}{c}{\alpha}^{+}\ast \delta (s)\ \left( if\ r(s)>0\right)\\ {}{\alpha}^{-}\ast \delta (s)\ \left( if\ r(s)<0\right)\end{array}\right. $$

This second model allows updates to occur differently according to whether a reward is received or not. We refer to the mean difference in learning rates as the *learning bias* (*α*^+^ − *α*^−^). A positive learning bias (*α*^+^ > *α*^−^) indicates that participants adjust their estimates of the reward rate to a greater extent when a reward is obtained compared to when rewards are absent. The converse is true when the learning bias is negative (*α*^+^ < *α*^−^). If there is no learning bias (*α*^+^ = *α*^−^), then this model is equivalent to the simpler Symmetric Model with a single α.

### Asymmetric Models with physiology

Next, we extended the Asymmetry Model described above to test whether learning from positive (*α*^+^) or negative (*α*^−^) information was modulated by trial to trial perturbations in the physiological state. For each physiological state (i.e., PEP and HR), we compared two separate models (i.e., four models were fitted in total).

For each physiological state, the first model (Asymmetry Phys *α*^+^) tested physiological modulation of the *α*^+^ parameter. In this model, *α*^+^ was adjusted at each moment in time, according to the participants physiological state on that trial:

8 $$ {\alpha}^{+}(s)={b}_0+{b}_1\ast phys(t) $$

Here, *t* indexes the current trial. *b*_1_ governs the extent to which the learning rate for positive information (*α*^+^) is adjusted by the trial-wise measure of the physiological state. *α*^−^ was unmodulated by physiological state in this model.

The second of these additional models (Asymmetry Phys *α*^−^) tested physiological modulation of the *α*^−^ parameter. This was setup exactly as for Asymmetry Phys *α*^+^, except *α*^−^ was adjusted on each trial, according to:

9 $$ {\alpha}^{-}(s)={b}_0+{b}_1\ast phys(t) $$

Here, *b*_1_ governs the extent to which the learning rate for negative information (*α*^−^) is adjusted on each trial. *α*^+^ was unmodulated by physiological state in this model.

In all models, the probability of capturing an item is estimated using a softmax choice rule, implemented at the final frame of the encounter screen as follows:

10 $$ P(capture)=\frac{1}{1+\mathit{\exp}\left({\beta}_0-{\beta}_1\left({r}_i-{c}_i\right)\right)} $$

This formulation frames the decision to accept an option as a stochastic decision rule in which participants (noisily) choose between two actions (capture/release) according to the value of each action. The temperature parameter *β*_1_ governs participants’ sensitivity to the difference between these two values whilst the bias term *β*_0_ captures a participant’s general tendency toward capture/release options (independent of the values of each action). Note that under the above formulation, negative values for *β*_0_ indicate a bias towards capturing options, positive values indicate a bias towards releasing options.

In each model, *ρ* was initialized at the beginning of the experiment to the arithmetic average reward rate across the experiment, but subsequently carried over between environments (Constantino & Daw, 2015; Garrett & Daw, 2019). For each participant, we estimated the free parameters of the model by maximizing the likelihood of their sequence of choices, jointly with group-level distributions over the entire population using an Expectation Maximization (EM) procedure (Huys et al., 2011) implemented in the Julia language (Bezanson, Karpinski, Shah, & Edelman, 2012) version 0.7.0. Models were compared by first computing unbiased marginal likelihoods for each subject via subject-level cross validation and then comparing these Leave-One-Out cross-validation (LOOcv) scores between models (e.g., Asymmetric vs. Symmetric) using paired-sample t-tests.

To formally test for differences in learning rates (*α*^+^, *α*^−^) we estimated the covariance matrix $\hat{\Sigma}$ over the group level parameters using the Hessian of the model likelihood (Oakes, 1999) and then used a contrast ${\mathrm{c}}^T\hat{\Sigma}\mathrm{c}$ to compute the standard error on the difference *α*^+^ - *α*^−^.

## Results - Analysis branch 2: Sympathetic stress and learning parameters

A key feature of the learning previously observed in this task (Garrett & Daw, 2019) is that individuals adjusted their subjective estimates of the reward rate to a greater degree when the update was in a positive compared to a negative direction. This *learning asymmetry* accounted for the order effect whereby participants changed capture rates between environments to a greater degree when richness improved (participants transitioned from downturn to boom) compared to when richness deteriorated (participants transitioned from boom to downturn), an effect we also observe here (see Fig. 1D).

First, we tested if the same learning asymmetry was present in our data by fitting choices to two reinforcement learning models (Sutton & Barto, 1998): a *Symmetric Model* and an *Asymmetric Model,* exactly as done previously (Garrett & Daw, 2019). Both models used a delta-rule running average (Rescorla & Wagner, 1972) to update *ρ* according to positive and negative prediction errors. Negative prediction errors were generated every second that elapsed without a reward (e.g., each second of a time delay). Positive prediction errors were generated on seconds immediately following a time delay when rewards were received. The difference between the Symmetric Model and the Asymmetric Model was whether there were one or two learning-rate parameters. The Symmetric Model contained just a single learning parameter, *α*. This meant that *ρ* updated at the same rate regardless of whether the update was in a positive or a negative direction. The Asymmetric Model had two learning parameters: *α*^+^ and *α*^−^. This enabled *ρ* to update at a different rate, according to whether the update was in a positive (*α*^+^) or a negative (*α*^−^) direction.

Replicating past findings (Garrett & Daw, 2019), the Asymmetric Model again provided a superior fit (see Table 1) to the choice data than the Symmetric Model (*t*(19) = 3.14, *p* = 0.005, paired-sample t-tests comparing LOOcv scores for the Asymmetry vs. the Symmetric Model) with information integration again being biased in a positive direction (*α*^+^ > *α*^−^ : *z* = 1.80, *p* < 0.05 one-tailed). Prediction errors that caused *ρ* to shift upwards (following receipt of a reward) had a greater impact than prediction errors that caused *ρ* to shift downwards (following the absence of a reward).

**Table 1:** Model fitting and parameters for Symmetry and Asymmetry Models. The table summarizes for each model its fitting performances and its average parameters

Model	LOOcv	α	α+	α-	β_0_	β_1_
Symmetry Model	1230.54	-2.49 [95% CI: ± 0.54]			1.80 [95% CI: ± 0.66]	0.15 [95% CI: ± 0.05]
Asymmetry Model	1076.02**	-	-2.17 [95% CI: ± 0.38]	-2.38 [95% CI: ± 0.49]	-0.15 [95% CI: ± 1.79]	0.13 [95% CI: ± 0.03]

*LOOcv* leave-one-out cross-validation scores, summed over participants, *α* learning rate for both positive and negative prediction errors (Symmetric Model), *α+* learning rate for positive prediction errors, *α-* average learning rate for negative prediction errors (Asymmetric Model), *β*_*0*_ softmax intercept (bias towards reject), *β*_*1*_ softmax slope (sensitivity to the difference in the value of rejecting versus the value of accepting an option). Data are expressed as mean and 95% confidence intervals (CIs) (calculated as 1.96*standard error). Note: learning rates displayed here (α, α+, and α-) are untransformed parameters from the model fitting procedure; the function 0.5 + 0.5*erf(α/sqrt(2)) is subsequently applied to transform these to conventional learning rates within the range 0–1 **p<0.01 comparing LOOcv scores between the two models, paired-sample t-test

Next, we looked to relate our fine-grained trial-wise measures of participants physiological state to learning from positive (*α*^+^) and negative (*α*^−^) prediction errors. To do this, we tested two further models: (1) Asymmetry Phys *α*^+^; (2) Asymmetry Phys *α*^−^, separately for PEP and HR. Each model respectively allowed *α*^+^ or *α*^−^ to change as a function of participants’ trial-to-trial physiological state (see *Methods* – *Analysis branch 2*). Results are summarized in Table 2; in the case of PEP, we did not find evidence that learning from positive prediction errors (*α*^+^) was modulated by its state (*p* = 0.135); however, we did find evidence that learning from negative prediction errors (*α*^−^) was modulated (*t*(19) = 2.18, *p* = 0.042; paired-sample t-tests comparing both Asymmetry PEP models vs. Asymmetry model). The direction of the effect in this model was such that negative prediction errors that caused *ρ* to shift downwards (following the absence of a reward) changed beliefs to a greater extent when sympathetic drive was high (shorter PEP). In other words, participants were faster to learn that their environment was deteriorating when the sympathetic branch of the autonomic state was heightened. In the case of HR, we observed no evidence that learning from either prediction error was modulated by the physiological state (both p-values above 0.222).

**Table 2:** Model fitting and parameters for physiology-modulated learning models. Each model allows either α+ or α- (untransformed) to be modulated on a trial by trial (t) basis according to one of the physiological readouts (PEP or HR) according to an intercept and a slope (e.g., α+(t) = intercept + w*PEP(t)). A transfer function [0.5 + 0.5*erf(α/sqrt(2))] is then used to convert this to a conventional learning rate. The alternate learning rate is not modulated by physiology and is fit as a single free parameter. The table summarizes for each model its fitting performances and its average parameters

Model	LOOcv	α+	Intercept (α+)	w(α+)	α-	Intercept (α-)	w(α-)	β_0_	β_1_
PEP Modulate α+	1023.63		-2.60 [95% CI: ± 1.23]	0.002 [95% CI: ± 0.02]	-2.67 [95% CI: ± 0.40]			-0.19 [95% CI: ± 1.88]	0.13 [95% CI: ± 0.03]
PEP Modulate α-	1008.02*	-2.41 [95% CI: ± 0.21]				-2.11 [95% CI: ± 1.05]	-0.007 [95% CI: ± 0.01]	-0.30 [95% CI: ± 1.83]	0.14 [95% CI: ± 0.03]
HR Modulate α+	1031.42		-2.05 [95% CI: ± 0.96]	-0.49 [95% CI: ± 1.25]	-2.71 [95% CI: ± 0.34]			-0.22 [95% CI: ± 1.79]	0.13 [95% CI: ± 0.04]
HR Modulate α-	1082.25	-2.14 [95% CI: ± 0.34]				-1.50 [95% CI: ± 0.83]	-1.09 [95% CI: ± 1.31]	-0.33 [95% CI: ±1.97]	0.13 [95% CI: ± 0.03]

*LOOcv*: leave-one-out cross-validation scores, summed over participants, *α+* learning rate for positive prediction errors (untransformed), *α-* average learning rate for negative prediction errors (untransformed), *intercept* unstandardized intercept for regressing learning rate (α+ or α-) against physiology, *w* unstandardized slope for regressing learning rate against physiology, *β*_*0*_ softmax intercept (bias towards reject), *β*_*1*_ softmax slope (sensitivity to the difference in the value of rejecting versus the value of accepting an option) Data are expressed as mean and 95% confidence intervals (CIs) (calculated as 1.96*standard error) *p<0.05 comparing LOOcv scores against scores for the basic Asymmetry Model, which does not include physiology measures (see Table 1), paired-sample t-test

## Methods - Analysis branch 3: Sympathetic stress and optimal behavior

Our analyses to this point reveal that perturbations in the reward rate are exclusively associated with changes in sympathetic tone, and that sympathetic activation increases learning symmetry between positive and negative information. We might accordingly expect sympathetic engagement to predict optimal performance in the prey selection task. Here we formalize optimal performance (*D* − *B Mid*) for each subject as the delta in the rate of mid-rank capture in the downturn environment, relative to the boom environment:

11 $$ D-B\  Mid=p{\left(\left\{2,3\right\}\right)}_{downturn}-p{\left(\left\{2,3\right\}\right)}_{boom} $$

where *p*(*X*)_*S*_ is the proportion of items in vector *X* captured in environment *S*. Higher positive *D* − *B Mid* values accordingly reflect more optimal prey selection in the downturn environment, which predicts better task performance. We also computed a similar downturn-boom delta value for each subject for both physiological variable:

12a $$ D-B\ {Drive}_{PEP}={\frac{\sum \limits_{t=k}^q PEP(t)}{q}}_{downturn}-{\frac{\sum \limits_{t=k}^q PEP(t)}{q}}_{boom} $$

12b $$ D-B\ {Drive}_{HR}={\frac{\sum \limits_{t=k}^q HR(t)}{q}}_{downturn}-{\frac{\sum \limits_{t=k}^q HR(t)}{q}}_{boom} $$

whereby each *D* − *B Drive* corresponds to the downturn-boom delta between the average of each trial-wise physiological state from trial *k* to *q*. Higher positive values reflect higher drive (i.e., increased contractility and increased HR) in the downturn environment. We probed the relationship between our assay of optimal performance and environment-wise physiology fluctuations with a linear model of each subject’s *D* − *B Mid* as a function of an intercept, and their *D* − *B Drive*_*PEP*_ and *D* − *B Drive*_*HR*_ values. Further, we ran two iterations of this model, one from the trial-wise measures taken from trials in the first half (i.e., 0–360 s) of the time spent in each environment (where we assumed the majority of learning is required) and as a control, another model using trials in the second half (360–720 s) of the time spent in each state. In other words, in Eq. [12(a-b)], for the first model *k* was always 1 and *q* was the last trial for each subject that started before 360 s. For the second model, *k* was the first trial that started after 360 s, and *q* was the subject’s final trial. Positive coefficients associate the engagement of the relevant physiological state with optimized learning.

## Results - Analysis branch 3: Sympathetic stress and optimal behavior

The linear model (see Fig. 3C) of *D* − *B Mid*, as a function of the *D* − *B Drive* value for both physiological states, estimated from trials in the first half (early) of each environment returned a significant positive coefficient for *D* − *B Drive*_*PEP*_ (*β* = 0.389, *s*. *e*. = 0.164, *p* = 0.030),with the coefficient for *D* − *B Drive*_*HR*_ failing to reach statistical significance (*β* =  − 0.193, *s*. *e*. = 0.324, *p* = 0.559). The model of *D* − *B Mid* using physiological *D* − *B Drive* values estimated from trials in the second half (late) of environments did not return any significant coefficients (both p-values > 0.176). These final models suggest that the sympathetic engagement during crucial learning periods of a low reward environment predicts optimal behavioral adjustment. (For a similar pattern of results using a larger number of smaller time bins, see Supplementary Materials).

## Discussion

Appraising sequential offers of reward relative to an unknown future opportunity that is coupled with a time cost requires an optimization policy that draws on a belief about the richness characteristics of the current environment. Across a range of experiments, including reinforcement-learning tasks, belief updating paradigms and prey selection, information integration shows a positive bias (Eil & Rao, 2011; Garrett & Daw, 2019; Garrett & Sharot, 2014, 2017; Garrett et al., 2014; Garrett, González-Garzón, Foulkes, Levita, & Sharot, 2018; Korn, Prehn, Park, Walter & Heekeren, 2012; Kuzmanovic, Jefferson, & Vogeley, 2015, 2016; Kuzmanovic & Rigoux, 2017; Lefebvre, Lebreton, Meyniel, Bourgeois-Gironde, & Palminteri, 2017). That is, the rate at which humans update their belief about a probability, association, or reward rate is more sluggish if the new information carries a negative or aversive valence or telegraphs a deterioration of the current belief. In our prey selection task, subjects updated their belief about the rate of harvested reward with a similar bias toward reward over delay information. By using simultaneous continuously recorded cardiac autonomic physiology measures, we reveal a uniquely adaptive role for the sympathetic branch in this situation.

In our choice models (analysis branch 1), the positive association between value and choice was principally associated with a reduced heart rate, whether or not the immediate context was factored into the value of the offer, i.e., whether cost reflected the objective capture-time of an invader and reward reflected the objective harvested fuel, or whether these value dimensions were collapsed into a single variable that compared reward to the opportunity cost of capture in the current reward context. In contrast, we observed contextual derivatives, i.e., changes in the rate of harvested reward having a unique association with sympathetic state derivatives. Specifically, increases in contractility (shorter PEP) scaled with decreases in the average rate of reward harvested per second. No relationship emerged between contractility changes and positive fluctuations in the environment’s richness, establishing that the sympathetic modification to environmental changes was isolated to negative valence. In our learning models (analysis branch 2), drive in the sympathetic system was uniquely associated with an increased rate of learning, and specifically when reward rate estimates were updated via negative prediction error. And finally, in analysis 3, we revealed that the unique relationship between sympathetic drive and learning was not exclusively a phenomenological response to a worsening environment, by observing a positive relationship between activation of the sympathetic state during crucial periods of the task – i.e., early in the downturn environment – and deployment of an optimal behavioral policy – i.e., increased capture of mid-rank invaders.

The only other study (Lenow et al., 2017) to probe the stress system in a human foraging task demonstrated an opposing relationship whereby stress increased overharvesting. Overharvesting occurs when an animal or human exploits proximal known resources beyond a threshold determining that better yields would be obtained by switching location. Such maladaptive perseveration indicates a biased low estimation of environmental quality. That this tendency is exacerbated by stress is interestingly both consistent and at odds with our finding that sympathetic stress adaptively increases the rate of negative information integration. Consistent, in so far as stress drives a pessimistic learning process in both cases, but at odds in terms of adaptivity. It could be the case that stress simply plays a general role in driving more pessimistic foraging behavior and whether or not this proves adaptive is an arbitrary consequence of task design. However, it’s important to also note that Lenow et al. (2017) assayed the hypothalamic-pituitary-adrenal (HPA) axis of the stress response via cortisol. It may be a step too far to directly compare these two studies, which characterize stress with different measures and on different time scales. Some studies show alignment between the sympathetic system and the HPA response to stressors (e.g., Bosch et al., 2009), others demonstrate task selectivity, particularly where subjects feel threat or a loss of control (see Dickerson & Kemeny, 2004, for a review) and others still argue a sequential framework that sees HPA respond once a threshold level of activation has been reached by the sympathetic system (Bosch et al., 2009; Cacioppo et al., 1995).

The unique reactivity of the sympathetic branch of the autonomic system for learning that the rate of reward is deteriorating is consistent with division specific cortical control of autonomic function. Meta-analysis of human neuroimaging data demonstrate that divergent brain networks regulate the sympathetic and parasympathetic branch, with control of the former including prefrontal and insular cortices, in addition to multiple areas within the medial wall of the mid and anterior cingulate (ACC; Beissner, Meissner, Bär, & Napadow, 2013; Dum, Levinthal, & Strick, 2016). Further, animal tracer evidence (Dum et al., 2016) reveals direct synaptic inputs into the adrenal medulla – a key sympathetic site for catecholaminergic release – from both pregenual and subgenual portions of ACC. Converging literature further points to ACC playing a key role in the learning and policy formation requirements in our prey selection task. Firstly, ACC is broadly considered a multi-faceted controller in goal-directed behavior, aligned with such general cognitive mechanisms as conflict monitoring (e.g., Botvinick, Nystrom, Fissell, Carter, & Cohen, 1999) and pre-emptively signaling the likelihood of an error (e.g., Carter et al., 1998) or reward (e.g., Hayden & Platt, 2010) of an action. ACC function has also been implicated more specifically in mechanisms relevant to foraging, such as signaling the amount of effort invested by animals in exchange for reward (Rudebeck, Walton, Smyth, Bannerman, & Rushworth, 2006; Walton et al., 2009), the decision by humans to explore alternative options (foraging choices) beyond an immediate offer (Kolling et al., 2012; Shenhav, Straccia Botvinick, & Cohen, 2016), or integrating delayed components of value into adaptive switches in choice behavior (Economides, Guitart-Masip, Kurth-Nelson, & Dolan, 2014). Relevant to our specific finding is evidence that ACC is recruited in decisions that involve costs or negative affect (Amemori & Graybiel, 2012; Shackman et al., 2011; Walton et al., 2009) and tracking reward history (Bernacchia, Seo, Lee, & Wang, 2011; Seo, Barraclough & Lee, 2007). Taken together, the specific mobilization of the sympathetic stress system during environmental deterioration may be a support mechanism for a number of extra demands being placed on ACC in this context.

The order effect we observe, and the learning account of it, replicates past findings (Garrett & Daw, 2019) and indicates that participants carry over information about the reward rate from one environment into the next. This is despite the fact that participants are explicitly told at the start of the experiment that they will experience different environments in the task and, during the experiment, new environments are clearly signaled. An important question that awaits future work is what impact increasing the frequency of switches has on the underlying learning process. For example, it may be the case that a greater number of transitions prompt a shift away from incrementally updating a single estimate of the environments reward rate towards maintaining and reinstating (previously learned) context specific reward rates over time. Future experiments will be required to test whether the learning process and its interaction with the sympathetic branch of the autonomic state varies with the number of reversals, potentially along with other factors such as time spent in each environment, the number of environments and contextual differences between environments.

While our data reveal an association between environmental deterioration and potentially adaptive drive in the sympathetic system, we still do not know the nature of this association’s underlying causal dynamics. Recent stereotactic electroencephalography evidence from a sample of epilepsy patients demonstrates that stimulation of the subgenual cingulate leads to a consistent and dramatic reduction in systolic, but not diastolic blood pressure, suggesting that this region can control the cardioinhibitory reduction of myocardial contractility (Lacuey et al., 2018). This finding converges with previous evidence of relationships between ventromedial prefrontal and subgenual cingulate cortices and electrodermal sympathetic tone (Nagai, Critchley, Featherstone, Trimble, & Dolan, 2004). However earlier positron emission tomography studies with pure autonomic failure patients (Critchley, Mathias, & Dolan, 2001) and studies correlating heart-rate variability with functional magnetic resonance imaging (fMRI) data in healthy subjects (Critchley et al., 2003) also implicate dorsal cingulate and insula cortex in generating cardiac autonomic arousal during mental effort (Radulescu, Nagai, & Critchley, 2015). Thus, while it’s likely that our observed sympathetic drive was elicited by cortical regions, the precise regions and dynamics remain to be established. Further, sympathetic recruitment may have been driven by regions outside of those typically implicated in decision making, for example, motor areas (primate tracer evidence highlights dense projections from ventral regions of medial motor areas to the adrenal medulla (Dum et al., 2016)). Thus, establishing whether task-specific nodes directly draw on sympathetic support via direct projections (brain-heart-brain), or whether both decision nodes and sympathetic support is recruited as part of an overarching network (brain-brain-heart) is an important future direction for research. Importantly, the ICG/ECG protocol used in this study can be used concurrently with both fMRI and high temporal resolution electroencephalography, allowing future studies to characterize both the substrates and temporal dynamics of cortical regions and sympathetic reaction in behavioral adaptation.

Our study used a cardiovascular measure (PEP) to probe activity in the sympathetic branch of the autonomic nervous system. PEP is considered the best available, non-invasive indicator of sympathetic impact on the heart, given its ability to directly index the force of beta-adrenergic myocardial contractility (Lewis, Leighton, & Forester, 1974; Light, 1985; Newlin & Levenson, 1979; Sherwood et al., 1986; Sherwood et al., 1990). PEP is largely insensitive to vagal tone (Linden, 1985) which further helps disambiguate findings from parasympathetic involvement - a confound with alternative cardiovascular measures such as low frequency heart rate variability (LF HRV; Berntson et al., 1997). Nonetheless, ascribing fluctuations in PEP to task-related (extrinsic) sympathetic drive is potentially compromised by additional local (intrinsic) effects. Specifically, PEP decreases with increased left ventricular filling (preload), and also with decreased aortic diastolic pressure (afterload; Newlin & Levenson, 1979; Obrist et al., 1987). We controlled for these local confounds by first correcting both the continuous ICG, and the trial-wise estimates of both PEP and HR for respiratory state, known to influence stroke volume/preload (Robotham et al., 1979). Second, we tested healthy stationary sitting young adults, instructed to minimize movement and posture change; maintaining a fixed sitting posture particularly helps to maintain consistent end-diastolic aortic pressure (afterload; Houtveen, Groot, & De Geus, 2005). Finally, HR, included in all of our models, provides a good estimate of ventricular filling; specifically, HR controlled for the Frank-Starling mechanism whereby beat-to-beat deceleration in heart rate, which increases preload, causes increased contractility (shorter PEP) for reasons not mediated by the sympathetic system (Kuipers et al., 2017; Sherwood et al., 1990). We are confident that the Frank-Starling effect does not apply to the primary association revealed by our learning models, models of reward rate derivatives, and model of choice optimization, all of which did not show concurrent influence of HR alongside PEP.

HR was uniquely found to scale negatively with low value capture. As predicted by MVT, and as evidenced by increases in mid-rank invader captures, the downturn environment increased the subjective value of lower value items. Thus, having learned of a deteriorated environmental richness (associated with increased sympathetic activation), subjects now require a change in behavioral policy. In other words, subjects must overwrite the prepotent policy of avoiding mid-rank invaders, and switch to a policy of capturing them. This raises the possibility that reduced heart rate is driven by increased executive control demands, recruiting parasympathetic engagement, in line with neurovisceral integration models of cognitive control (Thayer, Hansen, Saus-Rose, & Johnsen, 2009). Under this model, prefrontal-subcortical inhibitory circuits that govern the control of thoughts and goal-directed behavior provide inhibitory input to the heart via the vagus nerve (Benarroch, 1993; Ellis & Thayer, 2010). However, given the influence of sympathetic and parasympathetic stress on HR, this interpretation remains speculative. Future research would need to measure both sympathetic and parasympathetic variables on the same time scales, while participants perform prey selection in dynamic environments.

Expanding into other branches of autonomic stress is one promising avenue for future research, potentially also shedding light on the lack of HR effects in our main findings (recalling that both sympathetic drive and parasympathetic drive can modulate HR). Another important avenue of future research is to explore stress associations with other dimensions of foraging and decision-making. If sympathetic stress uniquely tracks the deteriorating reward rate of an environment, we first may see other stress responses (e.g., HR) selectively track mechanisms related to other environmental perturbations, such as threat. More broadly, future research should also probe whether adaptive sympathetic associations are observed in a foraging or decision task not requiring incrementally updating beliefs, such as binary decision making (e.g., Freidin & Kacelnik, 2011) or foraging in an immediately appraisable environment (e.g., Kolling et al., 2012). In addition, tasks could be developed to further probe the degree to which sympathetic stress specifically underscores learning environmental deterioration, or a broader learning framework in which participants shift from model-free heuristics (capture high-value items), to a more complex model-based policy that incorporates environmental factors (environmental richness) and longer time horizons into a more carefully considered behavioral policy (Korn & Bach, 2018). Model mediation would likely also recruit medio-frontal decision sites linked with the sympathetic stress system and would be largely independent from increased executive demands related to inhibiting action selection (which could be underscored by parasympathetic tone). These future research avenues can potentially map specific stress responses onto specific learning and decision mechanisms, in addition to charting their degree of adaptivity.

Cannon (1929) originally proposed that the body readies itself for “fight or flight” via secretions of the adrenal medulla, initiated by sympathetic neural projections from the thoracic spine. Our findings propose a role of the sympathetic system at an earlier point of deliberation in context specific decisions, which endures over the course of decision policy formation. Stress may not simply support increased motivation and vigor, and instead be more contextually and cognitively shaped.

## Electronic supplementary material

ESM 1 (DOCX 75 kb)
