# Supplementary material for: Sympathetic involvement in time-constrained sequential foraging
Source: Cogn Affect Behav Neurosci. 2020 May 27;20(4):730–45. doi: 10.3758/s13415-020-00799-0 (PMC7651516; doi:10.3758/s13415-020-00799-0)
Supplement: Supplementary file 1 — (DOCX 75 kb) [file 13415_2020_799_MOESM1_ESM.docx]

**Supplementary Materials**

**Analysis branch 1 – alternative ANOVA**

In analysis branch 1, we observed that foraging during the two order conditions differed only in terms of adjustments in the number of mid-rank captures in the downturn environment. Given that mid-rank captures involve two invader identities, we ran an additional ANOVA to see if similar results emerged, replacing the three level $rank$ variable (hi, intermediate, low), with a four level $rank$ variable that mapped onto the reward / cost combination of the four different invader identities (hi/low, hi/hi, low/low, low/hi). This ANOVA again reported a significant three-way interaction between $order$, $env$ and $rank$ ($F=3.39, df =\left( 3, 126 \right), p=0.020)$. Contrasting mean capture rates between BD and DB, for the eight levels of the $env$*$rank$ interaction, we fist observed significantly higher capture of hi/hi invaders in the downturn environment for order DB, relative to BD $(\mu=0.944$ vs. $\mu=0.341$, both $s.e. =0.080, p<0.001)$. In addition, we observed significantly higher capture of low/low invaders in the downturn environment for order DB, relative to BD $(\mu=0.709$ vs. $\mu=0.463$, both $s.e. =0.080, p<0.036)$. As with the original ANOVA, no other contrasts reached statistical significance. Foraging during the two order conditions accordingly differed in terms of increased capture rate of both mid-rank invader identities in the downturn environment.

**Analysis branch 1 - full model results**

| \| $ANOVA \sim choice$ \| \|  \|  \| \| --- \| --- \| --- \| --- \| \|  \| $df$ \| $F$ \| $p$ \| \| order \| (1,18) \| 5.63 \| 0.029 \| \| env \| (1,90) \| 14.2 \| <0.001 \| \| rank \| (2,90) \| 398 \| <0.001 \| \| order:env \| (1,90) \| 5.90 \| 0.017 \| \| order:rank \| (2,90) \| 11.1 \| <0.001 \| \| env:rank \| (2,90) \| 18.0 \| <0.001 \| \| order:env:rank \| (2,90) \| 3.97 \| 0.022 \| \|  \|  \|  \|  \| \| $LME \sim choice$ \|  \|  \|  \| \|  \| $\beta$ \| $s.e.$ \| $p$ \| \| intercept \| -0.173 \| 0.246 \| 0.483 \| \| reward(t) \| 2.07 \| 0.065 \| <0.001 \| \| delay(t) \| -2.05 \| 0.066 \| <0.001 \| \| reward(t-1) \| -0.287 \| 0.064 \| <0.001 \| \| delay(t-1) \| 0.071 \| 0.064 \| 0.265 \| \|  \|  \|  \|  \| \| $gLME \sim choice$ \|  \|  \|  \| \|  \| $\beta$ \| $s.e.$ \| $p$ \| \| intercept \| -1.51 \| 0.296 \| <0.001 \| \| reward \| 4.74 \| 0.151 \| <0.001 \| \| pep \| 0.334 \| 0.132 \| 0.011 \| \| delay \| -4.66 \| 0.151 \| <0.001 \| \| env \| 1.48 \| 0.123 \| <0.001 \| \| order \| 1.13 \| 0.398 \| 0.005 \| \| trial_index \| -0.368 \| 0.056 \| <0.001 \| \| pep:reward \| -0.200 \| 0.132 \| 0.128 \| \| pep:delay \| 0.461 \| 0.133 \| <0.001 \| \|  \|  \|  \|  \| \| $gLME \sim choice$ \|  \|  \|  \| \|  \| $\beta$ \| $s.e.$ \| $p$ \| \| intercept \| -1.48 \| 0.314 \| <0.001 \| \| reward \| 4.90 \| 0.163 \| <0.001 \| \| HR \| -0.068 \| 0.115 \| 0.554 \| \| delay \| -4.82 \| 0.163 \| <0.001 \| \| env \| 1.46 \| 0.122 \| <0.001 \| \| order \| 1.21 \| 0.426 \| 0.004 \| \| trial_index \| -0.415 \| 0.055 \| <0.001 \| \| HR:reward \| 0.922 \| 0.152 \| <0.001 \| \| HR:delay \| -0.703 \| 0.152 \| <0.001 \| \|  \|  \|  \|  \| \| $gLME \sim choice$ \|  \|  \|  \| \|  \| $\beta$ \| $s.e.$ \| $p$ \| \| intercept \| -0.236 \| 0.334 \| 0.481 \| \| value \| 3.95 \| 0.120 \| <0.001 \| \| pep \| 0.465 \| 0.128 \| <0.001 \| \| HR \| -0.013 \| 0.099 \| 0.893 \| \| env \| 0.607 \| 0.113 \| <0.001 \| \| order \| -0.708 \| 0.467 \| 0.129 \| \| trial_index \| -0.344 \| 0.056 \| <0.001 \| \| pep:value \| -0.188 \| 0.112 \| 0.092 \| \| HR:value \| 0.718 \| 0.108 \| <0.001 \| \|  \|  \|  \|  \| \| $LME \sim d\_mu$ \|  \|  \|  \| \|  \| $\beta$ \| $s.e.$ \| $p$ \| \| intercept \| 0.030 \| 0.005 \| <0.001 \| \| d_pep \| -0.008 \| 0.002 \| <0.001 \| \| d_HR \| -0.002 \| 0.002 \| 0.438 \| \| choice \| -0.017 \| 0.005 \| <0.001 \| \| trial_index \| -0.001 \| 0.002 \| 0.703 \| \| order \| 0.003 \| 0.005 \| 0.491 \| \| env \| -0.041 \| 0.005 \| <0.001 \| | |  |  |
| --- | --- | --- | --- | --- | --- | --- | --- | --- | --- | --- | --- | --- | --- | --- | --- | --- | --- | --- | --- | --- | --- | --- | --- | --- | --- | --- | --- | --- | --- | --- | --- | --- | --- | --- | --- | --- | --- | --- | --- | --- | --- | --- | --- | --- | --- | --- | --- | --- | --- | --- | --- | --- | --- | --- | --- | --- | --- | --- | --- | --- | --- | --- | --- | --- | --- | --- | --- | --- | --- | --- | --- | --- | --- | --- | --- | --- | --- | --- | --- | --- | --- | --- | --- | --- | --- | --- | --- | --- | --- | --- | --- | --- | --- | --- | --- | --- | --- | --- | --- | --- | --- | --- | --- | --- | --- | --- | --- | --- | --- | --- | --- | --- | --- | --- | --- | --- | --- | --- | --- | --- | --- | --- | --- | --- | --- | --- | --- | --- | --- | --- | --- | --- | --- | --- | --- | --- | --- | --- | --- | --- | --- | --- | --- | --- | --- | --- | --- | --- | --- | --- | --- | --- | --- | --- | --- | --- | --- | --- | --- | --- | --- | --- | --- | --- | --- | --- | --- | --- | --- | --- | --- | --- | --- | --- | --- | --- | --- | --- | --- | --- | --- | --- | --- | --- | --- | --- | --- | --- | --- | --- | --- | --- | --- | --- | --- | --- | --- | --- | --- | --- | --- | --- | --- | --- | --- | --- | --- | --- | --- | --- | --- | --- | --- | --- | --- | --- | --- | --- | --- | --- | --- | --- | --- | --- | --- | --- | --- | --- | --- | --- | --- | --- | --- | --- | --- | --- | --- | --- | --- | --- | --- | --- | --- | --- | --- | --- | --- | --- | --- | --- | --- | --- | --- | --- | --- |
|  | |  |  |
|  |  |  |  |
|  |  |  |  |
|  |  |  |  |
|  |  |  |  |
|  |  |  |  |
|  |  |  |  |
|  |  |  |  |
|  |  |  |  |
|  |  |  |  |
|  |  |  |  |
|  |  |  |  |
|  |  |  |  |
|  |  |  |  |
|  |  |  |  |
|  |  |  |  |
|  |  |  |  |
|  |  |  |  |
|  |  |  |  |
|  |  |  |  |
|  |  |  |  |
|  |  |  |  |
|  |  |  |  |
|  |  |  |  |
|  |  |  |  |
|  |  |  |  |
|  |  |  |  |
|  |  |  |  |
|  |  |  |  |
|  |  |  |  |
|  |  |  |  |

**Analysis branch 3 - alternative time binning**

In Analysis branch 3 we observe evidence that sympathetic engagement during crucial learning periods of a low reward environment predicts optimal behavioral adjustment. We ran two iterations of a model of behaviour optimisation variable $D-B Mid$ (see Analysis branch 3 methods), predicted by changes in PEP and HR during the first half (i.e. 0-360s) and second half (360-720s) of the blocks. Here we report a similar pattern of results, using an increased number of shorter time bins: quarter 1 (0-180s), quarter 2 (180-360s), quarter 3 (360-540s) and quarter 4 (540-720s). As summarized in the table below, only PEP approaches significance in the first two quarters, principally in the first.

|  | quarter1 | | quarter2 | | quarter3 | | quarter4 | |
| --- | --- | --- | --- | --- | --- | --- | --- | --- |
|  | $\beta$ $(se)$ | $p$ | $\beta$ $(se)$ | $p$ | $\beta$ $(se)$ | $p$ | $\beta$ $(se)$ | $p$ |
| PEP | 0.268 (0.137) | 0.067 | 0.294 (0.155) | 0.074 | 0.294 (0.184) | 0.129 | 0.214 (0.152) | 0.177 |
| HR | 0.115 (0.177) | 0.525 | -0.456 (0.296) | 0.141 | -0.283 (0.285) | 0.336 | -0.008 (0.188) | 0.965 |
